# The microbiologic spectrum of dacryocystitis

**DOI:** 10.1186/s12886-020-01792-4

**Published:** 2021-01-11

**Authors:** Ban Luo, Mu Li, Nan Xiang, Weikun Hu, Rong Liu, Xiaoqin Yan

**Affiliations:** 1 Department of Ophthalmology, Tongji Hospital, Tongji Medical College, Huazhong University of Science and Technology, 430030 Wuhan, China; 2 Department of Ophthalmology, Union Hospital, Tongji Medical College, Huazhong University of Science and Technology, 430022 Wuhan, China

**Keywords:** Microbiologic spectrum, Dacryocystitis, Adult and pediatrics, Acute and chronic

## Abstract

**Background:**

To investigate the microbiologic spectrum of dacryocystitis in adult and pediatric groups, specifically the microbiologic differences between chronic dacryocystitis with nasolacrimal duct obstruction (NLDO) and acute dacryocystitis in pediatric group.

**Methods:**

This retrospective study was reviewed for demographic and microbiologic profile of dacryocystitis. The culture results were reported.

**Results:**

Sixty-four adults and one hundred and five pediatrics with dacryocystitis were included in this study. Of all adults, only chronic dacryocystitis with NLDO was observed. Of all pediatric patients, 89 had chronic dacryocystitis with NLDO and 16 had acute dacryocystitis. Gram positive and negative isolates were numerically equal in adult group (both 36(48.65%)), while gram positive isolates were the major organism in pediatric group (71(58.68%)). *Streptococcus pneumonia* was the most common isolate in both adult (11(14.86%)) and pediatric (30(24.79%)) dacryocystitis. For both pediatric subgroups, gram positive isolates were the major organism (59(57.84%) for chronic dacryocystitis with NLDO and 12 (63.16%) for acute dacryocystitis). However, the leading isolates in those two subgroups were distinct, with *Streptococcus pneumonia* (29(28.43%)) being most common in chronic dacryocystitis with NLDO and *Staphylococcus aureus* (8(42.11%)) being most common in acute dacryocystitis.

**Conclusions:**

In adult group, gram negative isolates were more common in dacryocystitis than before. In pediatric group, gram positive isolates were still the major infection pathogen. Moreover, the more virulent organisms were more common in acute dacryocystitis than chronic dacryocystitis with NLDO.

## Background

Dacryocystitis is the most common disease of lacrimal drainage system. The reason for this infection is blockage of the lacrimal drainage system, which could further lead to the accumulation of tears and creation of a fertile environment for secondary bacterial infection, and dacryolith formation [1–3]. Bacterial dacryocystitis makes up about 60.8–94.9% of all dacryocystitis [4, 5]. Especially children with immature immune system could be more predispose to a severe infection [6]. The microorganisms reproduce in the lacrimal passage and result in the relevant symptoms, like epiphora, pyorrhea, ophthalmodynia and conjunctival hyperaemia, etc. Those symptoms would bring great discomfort to the patients and reduce their life quality. When not treated appropriately and timely, the infection might expand and lead to permanent injury of the lacrimal passage, orbital cellulitis, abscess, meningitis, cavernous sinus thrombosis, and even life-threatening situations [7–10]. In addition, the causes and the infection pathogen might also be distinct for chronic and acute dacryocystitis [11–14]. Thus, the understanding of microbiologic spectrum and control of microbiologic infection is critical and important in the treatment of dacryocystitis. However, there are relatively few studies focusing on the microbiologic characteristics of dacryocystitis and comparing the microbiologic difference between chronic and acute dacryocystitis [15].

Previous studies have reported that the pathogens in dacryocystitis were similar to those found in the upper respiratory passage and on the skin [2, 16]. However, the application of broad-spectrum antibiotics might have an influence on the microbiologic spectrum of dacryocystitis [17]. Although gram positive bacterium was reported to be predominant in most studies, some rarer gram negative bacteria and methicillin-resistant *Staphylococcus aureus* became more common in dacryocystitis recently [14, 15, 17, 18]. Patients with dacryocystitis often received empiric antibiotic treatment without or before the culture results, which need time to acquire [16]. Thus, changes in the microbiologic spectrum might obviously affect the therapeutic effect and outcome of dacryocystitis.

Accordingly, we performed this retrospective study to investigate the microbiologic spectrum of dacryocystitis in central China to provide microbiologic data for the clinical treatment of dacryocystitis.

## Methods

### Subjects

In this retrospective study, we included patients with dacryocystitis in the department of ophthalmology, Tongji hospital, Wuhan, China, from 2016.7 to 2017.7. This study was approved by the ethics committee of Tongji Hospital and performed in accordance with the tenets of the Declaration of Helsinki. Written informed consents were obtained from all participants. Patients were diagnosed as acute or chronic dacryocystitis based on their history, signs and symptoms. Chronic dacryocystitis was diagnosed as persistent epiphora and regurgitation of mucoid or mucopurulent material on pressure over the sac area or during irrigation of the lacrimal drainage system. Besides that, when the lacrimal sac area showed manifestation of pain, redness, and swelling, it would be diagnosed as acute dacryocystitis. Nasolacrimal duct obstruction (NLDO) was diagnosed according to the lacrimal passage irrigation test results. All cases of epiphora caused by lacrimal disease other than NLDO, patients with any history of maxillofacial surgery, maxillofacial trauma or maxillofacial tumor and patients who had received any topical or systemic antibiotics in the past one week ahead of their microbiologic culture were excluded [14].

### Sample collection and microbiologic culture

To collect the samples, a lacrimal probe would be placed to the lacrimal sac through the lower lacrimal ductile. Then the probe core within the probe would be removed. After that, a sterile syringe would be attached to the hollow lacrimal probe, to aspirate the lacrimal sac secretion. Once the samples were collected, they were sown immediately in transport medium. Transport medium were delivered to the laboratory within 15 minutes at room temperature. Samples from transport medium were planted in sheep blood agar, eosin methylene blue, USP althernative, Sabouraud’s dextrose agar and chocolate agar. Reproduction was checked intermittently. Clinically significant growths in samples taken from flora regions were reported. Strains were identified by manual biochemical reaction methods and/or instruments (VITEK-2-COMPACT system, bioMérieux, France and matrix-associated laser desorption ionization time-of-flight mass spectrometry, MALDI-TOF MS, Germany).

### Data analysis

The Chi-square test (SPSS software 19.0, IBM Corp., Armonk, NY, USA) was used for comparing ratio differences between groups. Statistical significance was defined as a *p* value of < 0.05.

## Results

### Subject characteristics

A total of 169 patients with dacryocystitis were included in this retrospective study. Of those patients, adult patients were 64 (37.87%) and pediatric patients were 105 (62.13%). In adult group, only chronic dacryocystitis with NLDO (64 (37.87%)) was observed. In pediatric group, both chronic dacryocystitis with NLDO and acute dacryocystitis were observed. Among them, 89 (52.66%) were patients with chronic dacryocystitis with NLDO, and 16 (9.47%) were patients with acute dacryocystitis. There were 80 (47.34%) male and 89 (52.66%) female patients. The age range was from 0 to 80 years old, with the mean age of 16.6 years old. Among the 169 patients, 21 (12.43%) of them had polymicrobial infections (Table 1).

**Table 1:** Study subject characteristics

Characteristics		Adult (%)	Pediatric (%)	Overall (%)
Number of patients	Chronic dacryocystitis with NLDO	64 (37.87%)	89 (52.66%)	153 (90.53%)
	Acute dacryocystitis	0	16 (9.47%)	16 (9.47%)
Number of patients	Male	18 (10.65%)	62 (36.69%)	80 (47.34%)
	Female	46 (27.22%)	43 (25.44%)	89 (52.66%)
Age range (years)		18–80	0–17	0–80
Mean age (years)		42.7	0.6	16.6
Number of patients with polymicrobial infections		9 (5.33%)	12 (7.10%)	21 (12.43%)

*NLDO* nasolacrimal duct obstruction

### The microbiologic spectrum of dacryocystitis in adult and pediatric groups

As summarized in Table 2, the total amount of adult samples was 74. Among them, gram positive and negative isolates were numerically equal (both were 36 (48.65%)), gram positive isolate were not predominant in adult group. The leading gram positive isolates in adult were *Streptococcus pneumoniae* (11 (14.86%)) and *Coagulase Negative Staphylococci* (9 (12.16%)). For gram negative isolates in adult, *Haemophilus influenza* was relatively more frequent (5 (6.76%)).

**Table 2:** The microbiologic spectrum of dacryocystitis in adult and pediatric groups

		Adults (%)	Pediatrics (%)	Total (%)
**Gram positive isolates**	***Streptococcus pneumoniae***	**11 (14.86%)**	**30 (24.79%)**	41 (21.03%)
	*Staphylococcus aureus*	6 (8.10%)	14 (11.57%)	20 (10.26%)
	*Staphylococcus epidermidis*	2 (2.70%)	2 (1.65%)	4 (2.05%)
	*Coagulase negative Staphylococci*	9 (12.16%)	3 (2.47%)	12 (6.15%)
	*Streptococcus oralis*	0	8 (6.61%)	8 (4.10%)
	*Viridans Streptococci*	5 (6.76%)	4 (3.31%)	9 (4.62%)
	*Other gram positive isolates*	3 (4.05%)	10 (8.26%)	13 (6.67%)
	***Gram positive isolates in total***	36 (48.65%)	71 (58.68%)	107 (54.87%)
**Gram negative isolates**	*Pseudomonas aeruginosa*	3 (4.05%)	4 (3.31%)	7 (3.59%)
	*Escherichia Coli*	3 (4.05%)	2 (1.65%)	5 (2.56%)
	*Moraxella catarrhalis*	4 (5.41%)	3 (2.47%)	7 (3.59%)
	***Haemophilus influenzae***	**5 (6.76%)**	**7 (5.78%)**	12(6.15%)
	*Haemophilus parainfluenzae*	0	2 (1.65%)	2 (1.03%)
	*Other gram negative isolates*	21 (28.38%)	14 (11.57%)	35 (17.95%)
	***Gram negative isolates in total***	36 (48.65%)	32 (26.45%)	68 (34.87%)
**Fungus** **isolates**	*Streptococcus mitis*	0	9 (7.43%)	9 (4.61%)
	*Candida Parapsilosis*	1 (1.35%)	7 (5.79%)	8 (4.10%)
	*Other fungus*	1 (1.35%)	2 (1.65%)	3 (1.54%)
	***Fungus in total***	2 (2.70%)	18 (14.88%)	20 (10.26%)
	***microorganisms in total***	74 (100%)	121 (100%)	195 (100%)

In pediatric group, the sample size was 121, which was much bigger than that of adult group (74 samples). Among them, more than half of the samples (71 (58.68%)) were tested to be gram positive isolates, which was equal to the proportion of gram positive isolates in adult group (48.65%, *p* = 0.172). The most common gram positive isolate in pediatric group was *Streptococcus pneumoniae* (30 (24.79%)), followed by *Staphylococcus aureus* (14 (11.57%)). Besides the gram positive isolates, the rest 32 (26.45%) and 18 (14.88%) samples were proven to be gram negative isolates and fungus isolates, respectively. The relatively more common gram negative isolate was *Haemophilus influenza* (7 (5.78%)). Compared with adult group, the gram negative isolates were significantly less common (36 (48.65%) vs. 32 (26.45%), *p* = 0.002) and the fungus samples were significantly more common (2 (2.70%) vs. 18 (14.88%), *p* = 0.007) in pediatric group.

### The microbiologic spectrum of dacryocystitis in pediatric subgroups

As shown in Table 3, in both pediatric subgroups, gram positive isolates accounted for more than half of the total amount of microorganisms. 59 (57.84%) isolates in chronic dacryocystitis with NLDO group and 12 (63.16%) isolates in acute dacryocystitis group were gram positive isolates, with no significant proportion difference between two subgroups (*p* = 0.666). Besides that, the gram negative isolates were also equal in proportion in both subgroups (26 (25.49%) vs. 6 (31.58%), *p* = 0.581).

**Table 3:** The microbiologic spectrum of dacryocystitis in pediatric subgroups

Pediatric group		Chronic dacryocystitis with NLDO (%)	Acute dacryocystitis (%)
**Gram positive isolates**	***Streptococcus pneumoniae***	**29 (28.43%)**	1 (5.26%)
	***Staphylococcus aureus***	6 (5.88%)	**8 (42.11%)**
	*Staphylococcus epidermidis*	2 (1.96%)	0
	*Coagulase negative Staphylococci*	1 (0.98%)	2 (10.53%)
	*Streptococcus oralis*	8 (7.88%)	0
	*Viridans Streptococci*	3 (2.94%)	1 (5.26%)
	*Other gram positive isolates*	10 (9.80%)	0
	***Gram positive isolates in total***	59 (57.84%)	12 (63.16%)
**Gram negative isolates**	*Pseudomonas aeruginosa*	3 (2.94%)	1 (5.26%)
	*Escherichia Coli*	2 (1.96%)	0
	*Moraxella catarrhalis*	3 (2.94%)	0
	*Haemophilus influenzae*	6 (5.88%)	1 (5.26%)
	*Haemophilus parainfluenzae*	2 (1.96%)	0
	*Other gram negative isolates*	10 (9.80%)	4 (21.05%)
	***Gram negative isolates in total***	26 (25.49%)	6 (31.58%)
**Fungus** **isolates**	*Streptococcus mitis*	9 (8.82%)	0
	*Candida Parapsilosis*	7 (6.86%)	0
	*Other fungus*	1 (0.98%)	1 (5.26%)
	***Fungus in total***	17 (16.67%)	1 (5.26%)
	***microorganisms in total***	102 (100%)	19 (100%)

*NLDO* nasolacrimal duct obstruction

For chronic dacryocystitis with NLDO group, the leading gram positive isolate was *Streptococcus pneumoniae* (29 (28.43%)). However, for acute dacryocystitis group, the leading gram positive isolate was not *Streptococcus pneumoniae* but *Staphylococcus aureus* (8 (42.11%)).

### The microbiologic spectrum of dacryocystitis in male and female groups

In the male group, nearly half of the collected samples were gram positive isolates (47 (48.96%)), with the leading isolate to be *Streptococcus pneumoniae* (16 (16.67%)). The rest samples were gram negative isolates (36 (37.50%)) and fungus (13 (13.54%)). In the female group, the results were similar to the male group. Among the total 99 samples, gram positive isolates occupied 60 samples (60.60%), which is equal to the proportion of that in male group (*p* = 0.102). The leading gram positive isolate was still *Streptococcus pneumoniae* (25 (25.25%)), followed by *Staphylococcus aureus* (14 (14.14%)). Moreover, the gram negative isolates were 32 (32.32%) and fungus samples were 7 (7.07%), and the proportions of gram negative isolates and fungus in both male and female groups were not significantly different (*p* = 0.448 and 0.136) (Table 4).

**Table 4:** The microbiologic spectrum of dacryocystitis in male and female groups

		Male (%)	Female (%)	Total (%)
**Gram positive isolates**	***Streptococcus pneumoniae***	**16 (16.67%)**	**25 (25.25%)**	41 (21.03%)
	*Staphylococcus aureus*	6 (6.25%)	14 (14.14%)	20 (10.26%)
	*Staphylococcus epidermidis*	3 (3.13%)	1 (1.01%)	4 (2.05%)
	*Coagulase negative Staphylococci*	4 (4.17%)	8 (8.08%)	12 (6.15%)
	*Streptococcus oralis*	6 (6.25%)	2 (2.02%)	8 (4.10%)
	*Viridans Streptococci*	1 (1.04%)	8 (8.08%)	9 (4.62%)
	*Other gram positive isolates*	11(11.45%)	2 (2.02%)	13 (6.67%)
	***Gram positive isolates in total***	47 (48.96%)	60 (60.60%)	107 (54.87%)
**Gram negative isolates**	*Pseudomonas aeruginosa*	3 (3.13%)	4 (4.04%)	7 (3.59%)
	*Escherichia Coli*	2 (2.08%)	3 (3.03%)	5 (2.56%)
	*Moraxella catarrhalis*	4 (4.17%)	3 (3.03%)	7 (3.59%)
	*Haemophilus influenzae*	8 (8.33%)	4 (4.04%)	12(6.15%)
	*Haemophilus parainfluenzae*	2 (2.08%)	0	2 (1.03%)
	*Other gram negative isolates*	17 (17.70%)	18 (18.18%)	35 (17.95%)
	***Gram negative isolates in total***	36 (37.50%)	32 (32.32%)	68 (34.87%)
**Fungus** **isolates**	*Streptococcus mitis*	7 (7.29%)	2 (2.02%)	9 (4.61%)
	*Candida Parapsilosis*	5 (5.21%)	3 (3.03%)	8 (4.10%)
	*Other fungus*	1 (1.04%)	2 (2.02%)	3 (1.54%)
	***Fungus in total***	13 (13.54%)	7 (7.07%)	20 (10.26%)
	***microorganisms in total***	96 (100%)	99 (100%)	195 (100%)

## Discussion

Dacryocystitis is mostly occurred with bacterial infections [4, 5], and might damage the normal structure of lacrimal duct [19]. In this retrospective study, we investigated the microbiologic culture results of dacryocystitis in adult and pediatric groups.

Similar to the endodontic infections, one microbiologic species could be nutrients for another one by the infections of lacrimal passage [16]. The polymicrobial infection rate of this study was 12.43% (21/169), which was equal to that of previous studies (7%-30%) [20, 21]. The average number of microorganisms was 1.15 per culture, which was lower than that of previous studies, with an average of 1.5–2.3 microorganisms per culture [14, 16, 22–24]. The reason for this difference might be that the majority (17/21) of polymicrobial infection cases of our study were with only two microorganisms.

The female-to-male ratio of this study was 1.11, which is significantly lower than the previous results [14–16]. The reason for this difference might be that the major (105/169) included subjects of this study were pediatrics, with overall mean age of 16.6 years old, while the previous studies included more adult than pediatrics in their studies, with mean age of from 44 to 60 years old [14–16]. Apart from pediatric group, the female-to-male ratio in adult group could increase to 2.56 in this study, which was consistent with the reported values of previous studies [14–16]. Contrary to the adult group, the female-to-male ratio in pediatric group was only 0.69, indicating that there were less female patients than male patients in pediatric group. Previously, very few studies have investigated the sex ratio of dacryocystitis patients in pediatric group with relatively big sample size. Our current results indicated that the sex ratio of dacryocystitis patients in pediatric and adult groups might be different. Unlike in adult group, female patients might not be predominant in the pediatric group.

In the adult group, no acute dacryocystitis were observed, while in the pediatric group, both acute dacryocystitis and chronic dacryocystitis with NLDO were found. Previous studies have also reported that younger patients were more susceptible to acute dacryocystitis than chronic dacryocystitis with NLDO [14, 25]. The immature immune system of children might be one reason for this phenomenon [6].

The predominant microbiologic spectrum (46–90%) of dacryocystitis were reported to be gram positive isolates, while gram negative isolates only constitute 2.5–40% of pathogens [13–16, 26–28]. Contrary to those previous studies, our adult group study results showed that the gram positive isolates were not predominant, and gram positive and negative isolates were numerically equal in adult group (both 36 (48.65%)), indicating that gram negative isolates became more common and took more proportion in infection pathogen of dacryocystitis than before in adult group. However, in pediatric group, the results were still similar to the previous studies [13–16, 26–28], with gram positive isolates being the major organism (71(58.68%)) and gram negative isolates only taking a small proportion (32 (26.45%)).

In adult group, the leading isolates were *Streptococcus pneumoniae* (14.86%) and *Coagulase negative staphylococci* (12.16%). In pediatric group, the leading isolates were *Streptococcus pneumoniae* (24.79%) and *Staphylococcus aureus* (11.57%). Thus, we could find that, in both adult and pediatric groups, *Streptococcus Pneumoniae* took the leading position, followed by *Staphylococcus* spp.. Those findings were similar to previous reports [17, 29, 30], implying that *Streptococcus pneumoniae* and *Staphylococcus* spp. were the most common isolates in dacryocystitis. Because *Streptococcus pneumonia* was a normal inhabitant of nasopharynx, immunization might be necessary for the restriction of its spreads to other sites (e.g., the ocular tissue and organ) [31]. For gram negative isolates, the most frequent isolate was *Haemophilus influenza* in both adult (5(6.76%)) and pediatric (7(5.78%)) groups, which was also consistent with previous reports [32, 33]. Besides that, compared with adult group, the fungus isolates were significantly more common in pediatric group (2 (2.70%) vs. 18 (14.88%), *p* = 0.007), implying that we should pay more attention to the anti-fungus treatment in pediatric dacryocystitis.

We divided the pediatric group into chronic dacryocystitis with NLDO and acute dacryocystitis groups, and found that the proportions of both gram positive and negative isolates were not significantly different between chronic and acute infection groups. However, the leading isolates in those two groups were distinct. For pediatric chronic dacryocystitis with NLDO, the leading isolates was *Streptococcus pneumonia* (28.43%), and for pediatric acute dacryocystitis, the leading isolates was *Staphylococcus aureus* (42.11%). Previous study has also suggested that the bacterial spectrum of acute and chronic dacryocystitis was different. The more virulent isolates (e.g., *Staphylococcus aureus*) might be more common by acute dacryocystitis [13, 14]. Previous study has reported that *Staphylococcus aureus* is the leading isolates of acute lacrimal infection in pediatric group [34], which was consistent with our results. We speculated that besides the immature immune system, the shorter and narrower nasolacrimal duct and the immature Hasner valve could also contribute to the more susceptibility to the more virulent pathogen in pediatric group. The more virulent pathogen could progress more rapidly and be more harmful to the tissue, leading to the acute dacryocystitis. Thus, the children are more prone to the acute dacryocystitis. However, we should also notice that this investigation was a single-center study conducted in the central China. Thus, the study results could have geographical and racial bias.

In term of sex, the isolates distribution showed no significant difference between male and female groups, with the leading isolate still to be *Streptococcus pneumonia*. Sex might have less influence on the microbiologic spectrum of dacryocystitis.

In this study, we used the lacrimal probe with sterile syringe at the end to collect samples from lacrimal sac. Using this noninvasive method, we could directly reach the lacrimal sac and take samples from lacrimal sac to the sterile syringe for culture. Thus, the collected samples would not expose to the conjunctival sac, avoiding the potential contamination by normal flora. Major normal flora of conjunctival sac has been confirmed to be gram positive isolates [2, 17, 35, 36]. Among them, *Staphylococcus epidermidis* accounts for about 57–87% of isolates, while *Streptococcus* spp. only occupied 6% [2, 17, 35, 36]. Meanwhile, our study showed that *Streptococcus pneumoniae* and *Staphylococcus aureus* were the most common isolates of dacryocystitis, which was different from the normal flora of conjunctival sac. Accordingly, we assume that the cultures obtained in this study was reliable, as there was a low possibility of contamination from conjunctival sac.

This study has certain limitations: First, this study was conducted in the central China, thus, our results might not be extrapolated to other geographical regions and races. Second, the incubation of microorganism relays on various conditions such as temperature, concentrations of carbon dioxide and incubation time. Thus, it’s possible that some microorganisms which are responsible for dacryocystitis were not isolated in current conditions. Third, sample collection is a complex process which can easily be contaminated, even though we improved the sample collection method and verified most of the isolated microorganism was pathogenic microorganism, but we could not exclude the possibility of contamination by colonized microorganisms.

## Conclusions

In adult dacryocystitis, gram positive and negative isolates were numerically equal to each other, indicating that gram negative isolates became more common in dacryocystitis than before [26–30, 36–40]. And *Streptococcus pneumonia* was still the most common organism in adult group. In pediatric dacryocystitis, gram positive isolates were much more common than gram negative isolates. *Streptococcus pneumonia* was the leading pathogen for chronic dacryocystitis with NLDO, and *Staphylococcus aureus* was the leading pathogen for acute dacryocystitis, indicating that the more virulent organisms were more common in acute dacryocystitis than chronic dacryocystitis with NLDO.

## Data Availability

The datasets used and analysed during the current study available from the corresponding author on reasonable request.

## Abbreviations

NLDO: Nasolacrimal duct obstruction
